# The Influence of Surface Processing on the Surface Plasmonic Enhancement of an Al-Nanoparticles-Enhanced ZnO UV Photodectector

**DOI:** 10.3390/nano13121877

**Published:** 2023-06-17

**Authors:** Gaoming Li, Qianwen Yan, Xiaolong Zhao, Yongning He

**Affiliations:** School of Microelectronics, Faculty of Electronic and Information Engineering, Xi’an Jiaotong University, Xi’an 710049, China; yanqw826@stu.xjtu.edu.cn (Q.Y.); zhaoxiaolong@xjtu.edu.cn (X.Z.); yongning@mail.xjtu.edu.cn (Y.H.)

**Keywords:** surface processing, localized surface plasmon, Al nanoparticles, ZnO UV photodetector

## Abstract

Surface Plasmonic Resonance (SPR) induced by metallic nanoparticles can be exploited to enhance the response of photodetectors (PD) to a large degree. Since the interface between metallic nanoparticles and semiconductors plays an important role in SPR, the magnitude of the enhancement is highly dependent on the morphology and roughness of the surface where the nanoparticles are distributed. In this work, we used mechanical polishing to produce different surface roughnesses for the ZnO film. Then, we exploited sputtering to fabricate Al nanoparticles on the ZnO film. The size and spacing of the Al nanoparticles were adjusted by sputtering power and time. Finally, we made a comparison among the PD with surface processing only, the Al-nanoparticles-enhanced PD, and the Al-nanoparticles-enhanced PD with surface processing. The results showed that increasing the surface roughness could enhance the photo response due to the augmentation of light scattering. More interestingly, the SPR induced by the Al nanoparticles could be strengthened by increasing the roughness. The responsivity could be enlarged by three orders of magnitude after we introduced surface roughness to boost the SPR. This work revealed the mechanism behind how surface roughness influences SPR enhancement. This provides new means for improving the photo responses of SPR-enhanced photodetectors.

## 1. Introduction

Ultraviolet photodetectors have wide applications in many fields, such as ozone layer monitoring, missile launch detection, flame sensing, UV radiation calibration and monitoring, high-speed optical communication, and astronomical research [1,2,3]. With the rapid progress of communication technology and the development of single-photon detection technology, improving the responsivity and sensitivity of detectors has become an important research aim recently. To this end, researchers have made many efforts, among which, the surface plasmon effect has been proven to be an effective way of improving the performance of UV photodetectors [4,5]. When the frequency of the incident light matches the resonant frequency of the metal’s free electrons at the sub-wavelength scale, the collective oscillation of the free electrons occurs, making the incident light become localized at the metal/dielectric interface, thus increasing the electromagnetic field strength and the scattering of the incident photons. This localized field enhancement can effectively increase the light absorption of semiconductors. The scattering enhancement will increase the optical path length within the semiconductor, thus greatly increasing the probability of incident photons being absorbed [6,7]. Localized surface plasmons (LSP) are preferred for another reason, which is that they can be directly excited by incident light without the need for considering wave vector matching [8].

ZnO is a widely used semiconductor, owing to its large bandgap, high exciton energy at room temperature, tunable conductivity, high transparency in the visible spectrum range, and good piezoelectric property. It has enormous applications in UV photodetectors [9,10,11], transparent conductive oxide [12,13], light-emitting diodes [14,15], thin film transistors [16,17], piezoelectric devices [18], nano-lasers [19], gas sensors [20], and photovoltaic cells [21]. We exploited ZnO as the active layer of UV photodetectors for its good photoelectric performance in the UV spectrum range. Actually, it has been demonstrated by a lot of research that its sensitivity can be greatly improved when the planar film is replaced by nanostructured materials, for the latter have large surface-volume ratios and a comparable Debye length to the size of the ZnO [22,23]. Of course, the size-controlled and reproducible synthesis of high-quality nanostructures is an important prerequisite to fabricating high-performance nano-devices [24].

Besides the various photodetectors based on nanostructures that have been reported, another technical route to improving responsivity is the use of surface plasmon enhancement. Metallic structures with subwavelength sizes are commonly used for SP (surface plasmon) enhancement. In recent years, studies on improving the performance of ultraviolet photodetectors using LSP have made some exciting progress. A variety of different metallic nanoparticles fabricated using various methods have been exploited to realize the enhancement of these PDs. In 2008, Zhou et al. [25] used a wet chemical method to embed Au nanoparticles with a size of about 150 nm into Ga_2_O_3_ nanowires, observing the surface plasmon resonance (SPR) behavior of the Au particles in the light-scattering spectrum [25,26,27]. Their light response ability was greatly improved under 532 nm illumination. The SPR effect occurred when the incident wave frequency matched the resonance frequency of the gold nanoparticles. In 2011, Duan’s research group [28] at UCLA obtained Au nanoparticles by annealing a 4 nm Au film on a SiO2 substrate and then transferring them onto graphene to make a photodetector. The Au nanoparticles served as a subwavelength scattering source and nano-antenna to enhance the light response at the plasma resonance frequency. Under 514 nm illumination, the photocurrent of the graphene detector integrated with the metal particles increased by at least four times. In addition, by changing the diameter of the Au nanoparticles, the response wavelength of the detector would also change. In 2014, Gao’s research group [29] prepared a ZnO nanowire array using chemical vapor deposition. Ag nanoparticles with a diameter of about 10 nm were fabricated on the surface of the ZnO nanowire through thermal evaporation. The modification of the Ag nanoparticles caused a blue shift in the UV emission peak of the ZnO nanowire. The photocurrent of the ZnO detector combined with the Ag particles increased two times under 365 nm light, with the response speed also being improved. In 2016, our research team prepared a ZnO-based UV detector embedded with Ag nanoparticles [30]. We prepared a solution of Ag nanoparticles through a chemical reduction method and spin-coated it onto ZnO thin films. Ag nanoparticles with a diameter of approximately 50 nm were successfully prepared. Under 365 nm light, the device’s responsivity was increased from 472 mA/W to 10.522 A/W. In 2017, Shen et al. [31] prepared a ZnO ultraviolet photodetector enhanced by Ag nanoparticles. They successfully increased the responsivity at 380 nm from 2.16 A/W to 2.86 A/W, while suppressing the responses at other wavelengths. Furthermore, the extinction and PL spectra showed that the enhancement of the response peak at 380 nm was due to the strong incident light scattering caused by the quadrupole plasmon resonance of the Ag nanoparticles. The suppression of the other wavelength responses was related to the shading effect of the Ag particles and surface defect passivation. In 2019, Khan et al. [32] modified the surface of ZnO nanorod array detectors with Au nanoparticles. Compared to bare ZnO nanorod photodetectors, the photocurrent of the Au-modified ZnO devices was significantly increased and their responsivity was increased by about four times under UV illumination.

Most work has focused on the modulation of the SPR by changing the metal type, size, spacing, and shape of the nanostructures. Actually, since the interface between the metallic structure and its surrounding layer is important for generating SPR, changing the surface morphology and roughness just provides another interesting dimension for tuning SPR. The relationship between surface morphology, roughness, and SPR enhancement is unclear as of yet. Moreover, the amplitude of the change in the roughness is critical to tuning the SPR. What is the proper value of roughness needed if we want to realize a higher responsivity after combining SP enhancement with surface roughness modulation? In this work, we found that semiconductor surface morphology and roughness would affect SPR enhancement. We realized an even greater responsivity after we introduced a proper change in the surface roughness to the SP-enhanced photodetector. We prepared Al nanoparticles using sputtering and tuned the size and spacing of the nanoparticles. The choice of Al instead of noble metals, such as Ag or Au, was mainly due to their low cost and potential of their application in the deep UV region. Actually, Al is a promising metallic material for UV plasmonics due to its relatively high quality factor in the UV range. We introduced surface roughness using mechanical polishing, which we refer to as surface processing elsewhere in this paper. The mechanism of how the surface morphology and roughness influenced the LSP was systematically studied using experiments and simulations. This provides a new means for the modulation of SPR and improving the performance using SPR.

## 2. Materials and Methods

### 2.1. Preparation and Surface Processing of ZnO Thin Films

In this study, ZnO thin films were prepared using RF magnetron sputtering. The sputtering equipment is GJP-450 (Institute of Microelectronics of the Chinese Academy of Sciences, Beijing, China). This sputtering system consisted of a sputtering chamber, vacuum pump, vacuum gauge, DC and RF power subsystem, gas subsystem, and heating subsystem. It was capable of sputtering metal, ceramic, and dielectric films. The sputtering velocity was adjusted mainly by power. The sputtering target had a diameter of 3 inches and a thickness of 5 mm. The surface processing was carried out using a Mecatech 234 automatic polishing machine (PRESI, Grenoble, France). The hardness of SiO_2_ in the SiO_2_-polishing solution was 6–7, close to that of ZnO. The AR90006 SiO_2_-polishing solution from the Goral Company (Dongguan, China) was selected for the polishing (SiO_2_ content was 38–41% and average particle size was 60 nm, and a Dac silk polishing cloth (Struers, Copenhagen, Denmark) (abrasive particle size was 1–9 μm) was selected as the polishing cloth. The ZnO film samples were polished following cold mounting with acrylic resin glue. The pressure, polishing head speed, polishing disc speed, and polishing duration were the key polishing parameters [33,34]. Finally, the polishing pressure was set to be 2 psi, the polishing head and disc speeds were both 20 rpm, and the polishing times were 20 s, 40 s, and 60 s.

### 2.2. Preparation of Al Nanoparticles and ZnO Photodetectors

**Figure 1:**
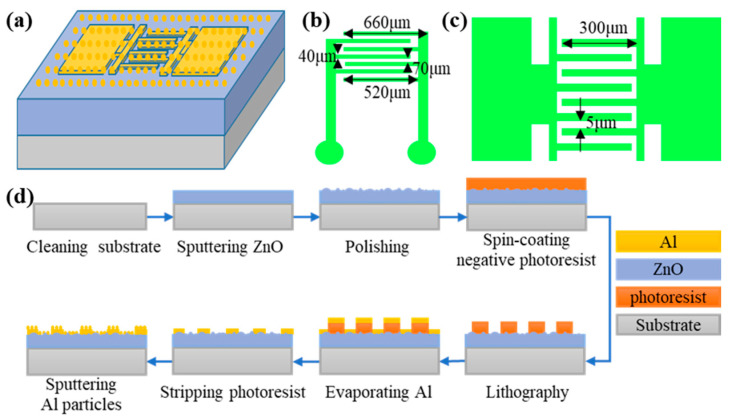
Structure diagram and preparation process of ZnO photodetectors with surface processing and Al nanoparticles. (**a**) Schematic diagram of device structure. Interdigitated electrode structure for (**b**) devices with Al nanoparticles; and (**c**) devices with surface processing. (**d**) Device preparation process.

The schematic structure of the ZnO photodetector with integrated Al particles is shown in Figure 1a, and the schematic diagrams of the interdigitated electrodes employed in this study are shown in Figure 1b,c. The preparation process for the ZnO photodetector integrated with Al nanoparticles is shown in Figure 1d. Firstly, the glass substrate was ultrasonically cleaned with acetone, ethanol, and deionized water in turn, then dried with N_2_ gas. Secondly, the ZnO thin films were prepared using RF magnetron sputtering. The sputtering time was 30 min, the sputtering pressure was 1 Pa, and the oxygen argon ratio was 5 sccm:10 sccm. Thirdly, the sample was cold embedded for the surface processing, followed by acetone immersion to remove the cold-embedded resin, and the sample was cleaned. The polishing time would affect the surface morphology and roughness. Fourthly, an NR9-3000PY negative photoresist (Futurrex, Franklin, NJ, USA) was spin-coated onto the surface of the sample, and then the coated substrate was baked (baking temperature was 140 °C and time was 1 min). Fifthly, the photoresist was patterned using photolithography: the exposure time was 120 s, the baking temperature was 110 °C, the baking time was 1 min, and the developer was DPD-200 (DONGJIN SEMICHEM, Seoul, South Korea). Sixthly, the Al metal layer was evaporated as electrodes using thermal evaporation. Seventhly, the sample was immersed in acetone and the remaining photoresist was removed. At this moment, the ZnO photodetector without Al particles was prepared. Finally, the Al nanoparticles were prepared on the device surface using sputtering, and the morphologies and sizes of the Al particles were adjusted by changing the sputtering power and sputtering time. The sputtering power range was from 80 W to 120 W. Generally speaking, the average size of the nanoparticles would increase with the sputtering power for a higher sputtering velocity, or we could increase the sputtering time to increase the size of the nanoparticles. The size dispersion of the nanoparticles was characterized using SEM (Scanning electron microsocopy). It did not show a notable change with the sputtering power and time based on the statistical analysis.

### 2.3. Testing, Characterization, and Simulation

We used the Gemini-500 scanning electron microscope (Carl Zeiss AG, Oberkochen, Germany) to characterize the surface morphology of the Al nanoparticles. The roughness of the ZnO thin films was measured using the Asylum MFP-3D Infinity type atomic force microscope (Asylum Research, Santa Barbara, CA, USA). The WFH-204B portable ultraviolet analyzer (Hangzhou Qiwei Instrument Co., Hangzhou, China) was used to provide a 365 nm ultraviolet light source and the Agilent B2902A dual channel source meter (Agilent, Santa Clara, CA, USA) was used to test the I-V characteristics. We used the Comsol multiphysics simulation tool to carry out the simulation. The incident light was along the z direction and the nanoparticles, which we treated as spheres, were placed on the ZnO film. The ZnO film was parallel to the x-y plane. We set a periodic boundary condition in both the x and y directions. The source port and exit port were at the top and bottom, respectively. We performed a parameter scan for the diameter from 20 nm to 35 nm and the spacing in between was 5 nm. The absorption and electric field distribution were obtained via a simulation.

## 3. Results and Discussion

### 3.1. Surface Morphology Characterization

#### 3.1.1. Effect of Surface Processing on the Morphology of ZnO

**Figure 2:**
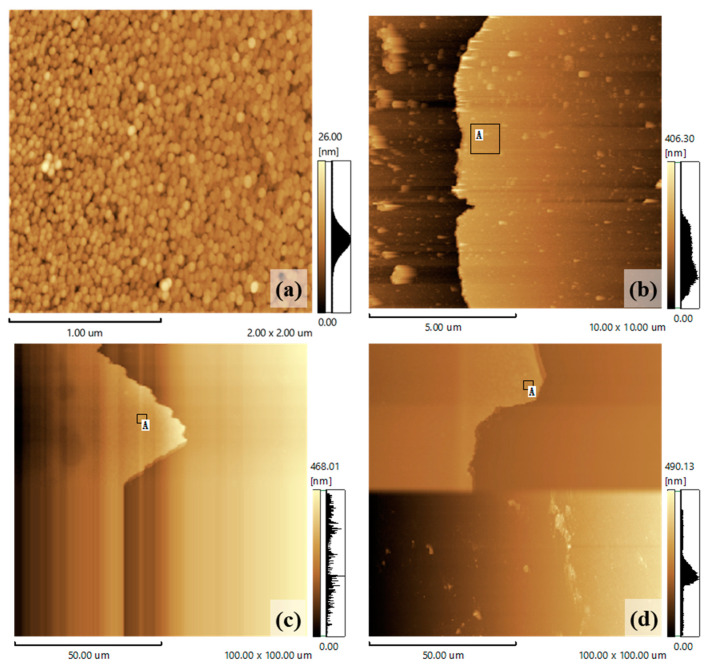
AFM of ZnO under different surface processing parameters: (**a**) unprocessed ZnO; (**b**) ZnO after processing for 20 s; (**c**) ZnO after processing for 40 s; and (**d**) ZnO after processing for 60 s. The black rectangular frame marked by letter A shows the area we used to calculate the roughness.

An AFM characterization was performed on the surface-processed ZnO samples. The influence of the surface processing on their surface morphology was studied by measuring the thickness and roughness of the ZnO region. Figure 2 shows the AFM images of the ZnO under different surface processing parameters. Figure 2a shows the morphology of the ZnO without surface processing and Figure 2b–d show the corresponding ZnO morphologies at surface processing times of 20 s, 40 s, and 60 s, respectively. We used a 1 μm × 1 μm area, which is indicated by a black rectangular frame and marked with the letter A to calculate the roughness. This step was used to measure the thickness of the ZnO film after the surface polishing. Table 1 shows the surface roughness and thickness of the ZnO under the different processing parameters. It can be observed that, with an increase in the surface processing time, the thickness of the ZnO decreased, and the surface roughness increased at first and then decreased.

#### 3.1.2. Tuning the Size and Spacing of Al Particles by Sputtering Power and Time

**Figure 3:**
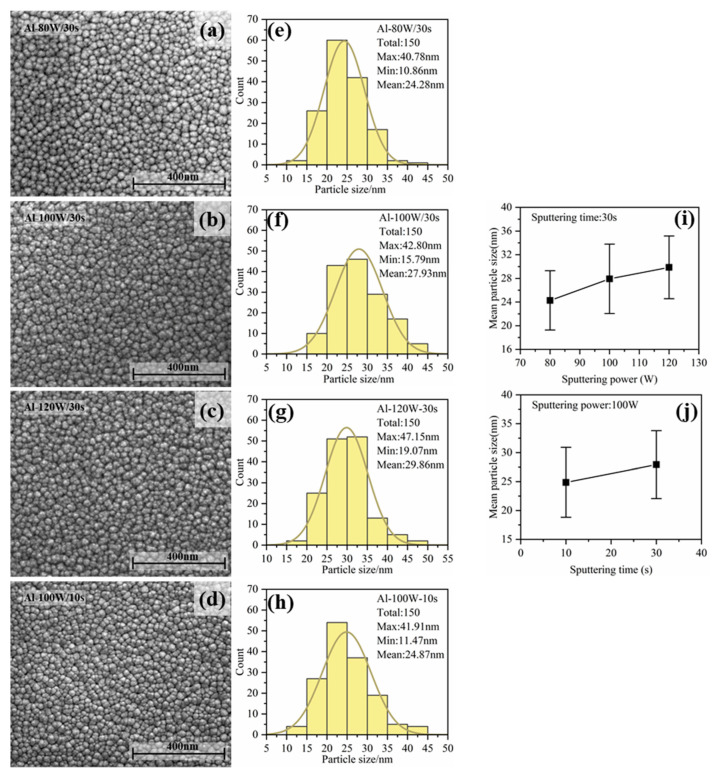
SEM of Al particles fabricated under different sputtering conditions: (**a**) sputtering power is 80 W, sputtering time is 30 s; (**b**) the sputtering power is 100 W and the sputtering time is 30 s; (**c**) the sputtering power is 120 W and the sputtering time is 30 s; and (**d**) sputtering time is 10 s, sputtering power is 100 W. The size distribution of NPs with sputtering conditions of (**e**) 80 W, 30 s, (**f**) 100 W, 30 s, (**g**) 120 W, 30 s, and (**h**) 100 W, 10 s. (**i**) The variation trend of average particle size with sputtering power; and (**j**) the variation trend of average particle size with sputtering time.

The SEM images of the Al particles fabricated under different sputtering conditions are shown in Figure 3. Figure 3a–c show the morphologies of the Al particles fabricated with sputtering powers of 80 W, 100 W, and 120 W (sputtering time of 30 s), respectively. Figure 3d shows the morphology of the Al particles fabricated with a sputtering power of 100 W and sputtering time of 10 s. The average particle size was analyzed statistically and the distribution of these sizes are shown in Figure 3e–h. The deviation of size was about 5 nm. Figure 3i,j show how the particles’ sizes changed with the sputtering power and time, respectively. Based on the mechanism of film growth, there were several stages, including nucleation, nucleated islands growing, and islands coalescence into a continuous film during the kinetic growth process. In our experiment, we needed to achieve the deposition of the Al nanoparticles within the first two stages. With an increase in the sputtering power and sputtering time, the average diameter of the Al particles showed an increasing trend. The main reason for this was that increasing the sputtering power usually caused a faster sputtering rate, so more Al atoms and clusters would deposit on the surface of the ZnO and aggregate into larger particles. If the sputtering rate was too high, the sputtered Al atoms and clusters would not have time to migrate on the surface of the ZnO, making them prone to forming a continuous Al film, which would not bring about the LSP effect. Similarly, Al particles would gradually accumulate with the sputtering time, leading to particle growth. Therefore, increasing the sputtering power and time would cause an increase in the size of the Al particles and a decrease in the spacing.

### 3.2. Photoresponse Testing and Analysis

#### 3.2.1. Effect of Surface Processing on the Performance of ZnO Photodetector

**Figure 4:**
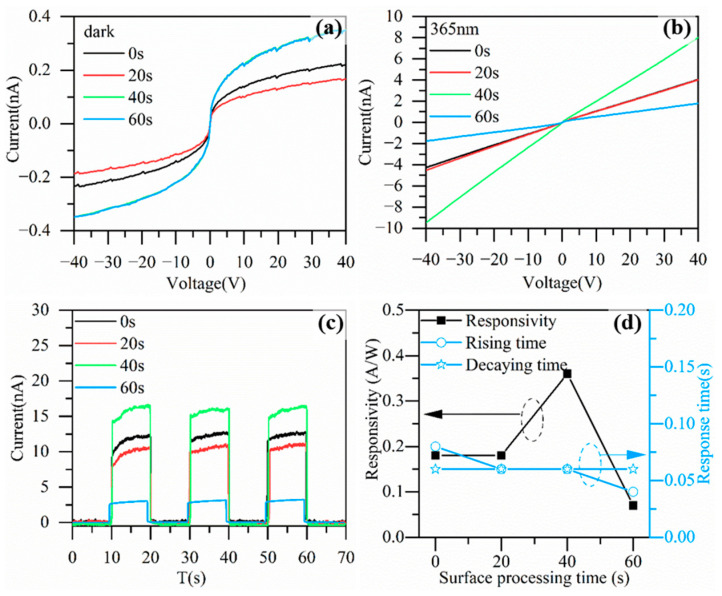
I-V characteristics of devices with different surface processing times. (**a**) Dark current, and (**b**) photocurrent at 365 nm. (**c**) I-T characteristics of devices at 40 V. (**d**) The dependence of responsivity, rising time, and decay time on surface processing time at 40 V.

The electrodes shown in Figure 1c were prepared on the surface-processed ZnO film, and the I-V characteristic curves of the devices with different surface processing parameters were obtained by applying scanning voltages of −40 V–40 V under dark and 365 nm light illumination, respectively. Figure 4a shows the dark currents. Processing for 40 s made the dark current increase by less than 200 pA at ±40 V, while processing for 20 s reduced the dark current by about 20 pA. Figure 4b shows the currents under 365 nm illumination, from which we can observe a considerable increase in the photocurrent after the surface processing, especially for the device with the ZnO film being processed for 40 s. Figure 4c shows the I-T characteristics measured at a voltage of 40 V. The devices were periodically irradiated with 365 nm ultraviolet light and the light-on state was 10 s within a cycle, with each cycle lasting for 20 s. Figure 4d shows the variation in the responsivity and response time with the surface processing time at a voltage of 40 V. The photocurrent and dark current levels of the processed 20 s device were close to those of the unprocessed device, with responsivities of 0.18 A/W. The photocurrent of the processed 40 s device increased dramatically, with a responsivity of 0.36 A/W. However, after processing for 60 s, the photocurrent of the corresponding device significantly decreased compared to the photocurrent of the unprocessed device, with a responsivity of only 0.07 A/W. In addition, surface processing did not show a notable impact on the response time of the device, as the rising and decay times were all less than 0.1 s and no prominent, changing trend was observed. In summary, the optical responsivity showed a trend of first increasing and then decreasing with the surface processing time. This was because the surface processing increased the surface roughness of the ZnO thin films, then increased the specific surface area of the ZnO and optical scattering to the incident photons in the ZnO, finally increasing the probability of the incident photons being absorbed by the ZnO. However, if the processing time was too long, it would cause severe damage to the ZnO thin films and the thickness of the ZnO thin films would significantly decrease. The photoresponse of the ZnO became worse due to the damage and defects introduced by over polishing. Based on the experimental results, it is considered that a processing time of around 40 s is optimal for improving the device performance, and subsequent experiments were also based on this processing parameter.

#### 3.2.2. Effect of Al Nanoparticles on the Performance of ZnO Photodetector

**Figure 5:**
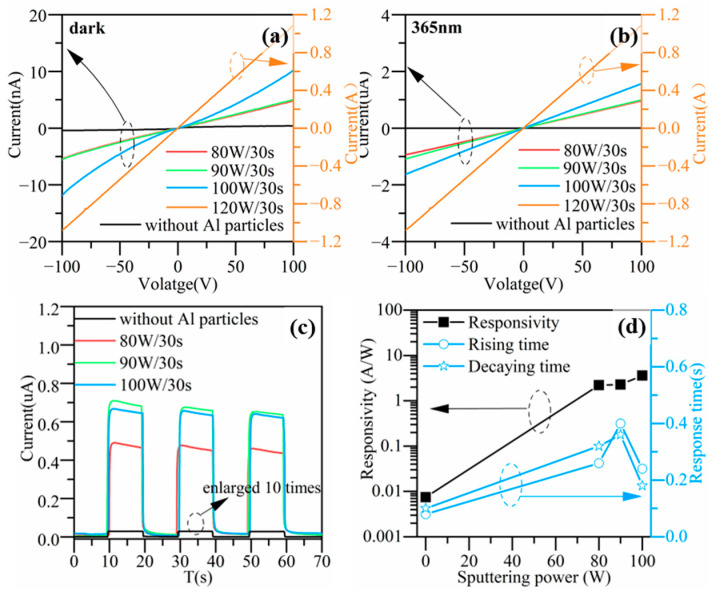
I-V characteristics of devices corresponding to different sputtering powers for Al nanoparticles (**a**) in dark, and (**b**) under 365 nm UV illumination. (**c**) I-T characteristics of devices at 100 V, data for the device without Al particles were enlarged by 10 times. (**d**) The dependence of responsivity, rising time and decay time of device on sputtering power at 100 V.

The ZnO photodetectors enhanced with Al nanoparticles prepared with different sputtering powers were tested in I-V and I-T measurements in dark and under 365 nm illumination. Figure 5a,b show the I-V characteristics of the devices corresponding to different Al sputtering powers. Figure 5c shows the I-T characteristics of the devices measured at a voltage of 100 V. The effect of the Al sputtering power on the device responsivity and response time is shown in Figure 5d. From the test results, it can be seen that the introduction of Al particles significantly increased the photocurrrent and dark current. With an increase in the Al sputtering power, the photocurrent, dark current, and responsivity increased and the increasing amplitude was greater with a larger sputtering power. At a sputtering power of 100 W, the responsivity achieved the maximum improvement of 481 times. When the Al sputtering power further increased to 120 W, the dark current increased significantly. Compared to the device without Al particles, the dark current increased by nine orders of magnitude and the I-V curves in dark and under UV illumination almost coincided with each other, indicating no light response. Therefore, it can be inferred that the Al particles sputtered at 120 W may have been too densely distributed, forming a continuous thin film. The device electrodes were short circuited by the continuous Al film. In addition, the response time slightly increased when the device was incorporated with Al particles.

**Figure 6:**
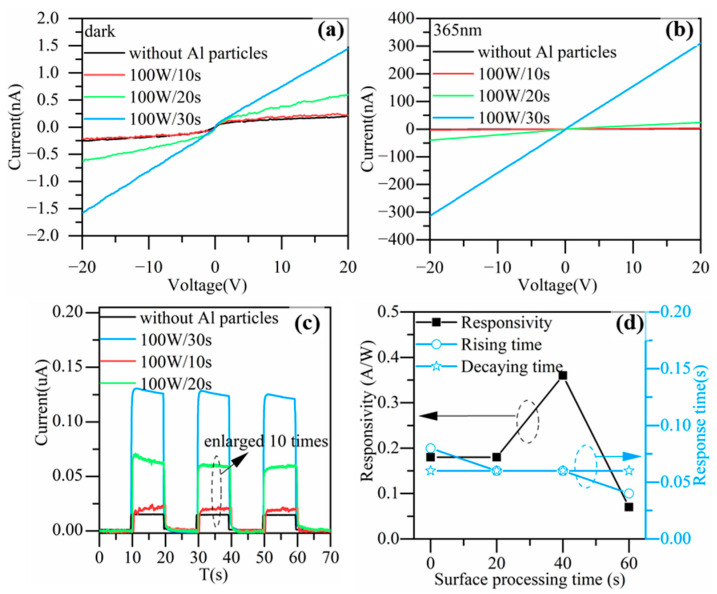
I-V characteristics of devices corresponding to different sputtering times for Al nanoparticles (**a**) in the dark, and (**b**) under 365 nm UV illumination. (**c**) I-T characteristics of devices at 20 V, part of data was enlarged by 10 times for observation. (**d**) The dependence of responsivity, rising time, and decay time of device on sputtering time at 20 V.

Next, we maintained a sputtering power of 100 W and further changed the sputtering time. I-V and I-T tests were carried out under dark and 365nm illumination, respectively. Figure 6a,b show the I-V characteristics of the devices corresponding to different sputtering times for the Al nanoparticles in the dark and under UV illumination. Compared to the device without Al nanoparticles, the dark currents of the devices with Al nanoparticles increased to a certain extent and the photocurrents also increased significantly. As the sputtering time increased, the photocurrent and dark current showed an increasing trend, and the increasing amplitude became larger with a longer sputtering time. Therefore, we can observe that the slope of the responsivity curve in Figure 6d increased. Figure 6c shows the I-T characteristics measured at a voltage of 20 V and Figure 6d shows the responsivity and rising and decay times at a voltage of 20 V. As the sputtering time increased, the responsivity of the device increased, and the response speed of the devices showed a trend of slowing down.

Based on the test results, it can be found that the introduction of Al nanoparticles would increase the dark current and response time. We attribute this increase in the dark current to some surface defects and trace Al doping related to the Al nanoparticles sputtering. In the process of Al particles sputtering, the sputtered Al atoms and clusters would collide with the surface of the ZnO film with a certain amount of energy, so this may have induced some defects on the surface and trace Al doping in the shallow layer near the surface. The surface defects and Al impurities would provide charge carriers to increase the dark current; this phenomenon has also been reported in the literature [35,36]. Moreover, the surface defect could slow down the response speed for the trapping and releasing of charge carriers [37]. The greater the power and longer the time we sputtered with, the probability of inducing surface defects and trace Al doping was higher.

**Figure 7:**
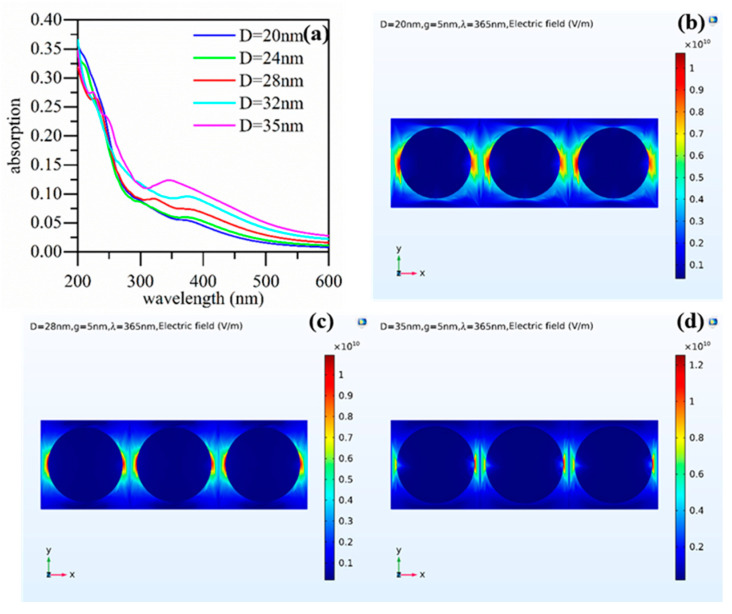
(**a**) Absorption spectra for Al nanoparticles with different diameters. Electric field distribution around the nanoparticles with a diameter of 10 nm (**b**), 30 nm (**c**), and 50 nm (**d**).

More importantly, the introduction of Al particles significantly improved the responsivity. This could be attributed to two major reasons. Firstly, the scattering of incident light by the Al nanoparticles increased the optical path length in the ZnO, thereby enhancing its light absorption; secondly, the Al nanoparticles produced the LSP effect, enhancing the electromagnetic field, increasing the photo-generated electron hole pairs [38,39]. With an increase in the sputtering time and sputtering power, the average diameter of the Al nanoparticles showed an increasing trend and the responsivity also increased. The simulation results shown in Figure 7 can explain this. Figure 7a shows the absorption spectra when the spacing of the Al nanoparticles was 5 nm and the diameter increased from 20 nm to 35 nm. As the diameter increased, the absorption gradually increased. Figure 7b–d show the electric field distribution around the Al nanoparticles with diameters of 20 nm, 28 nm, and 35 nm, respectively. The larger the size of the nanoparticles, the stronger the coupling of the electric field between the nanoparticles. Seen from the simulation results, nanoparticles with a larger size would cause more absorption of UV light at 365 nm. Usually, the spacing between would decrease with an increasing diameter and would also enhance the coupling between the neighboring particles. The stronger coupling between nanoparticles with a larger size would strengthen the absorption further. In addition, a larger nanoparticle always has a larger absorption cross-section and greater radiant intensity. As a result, the absorption peak would increase with the particle size [40,41].

#### 3.2.3. Effect of Surface Processing on the Performance of ZnO Photodetector Integrated with Al Nanoparticles

**Figure 8:**
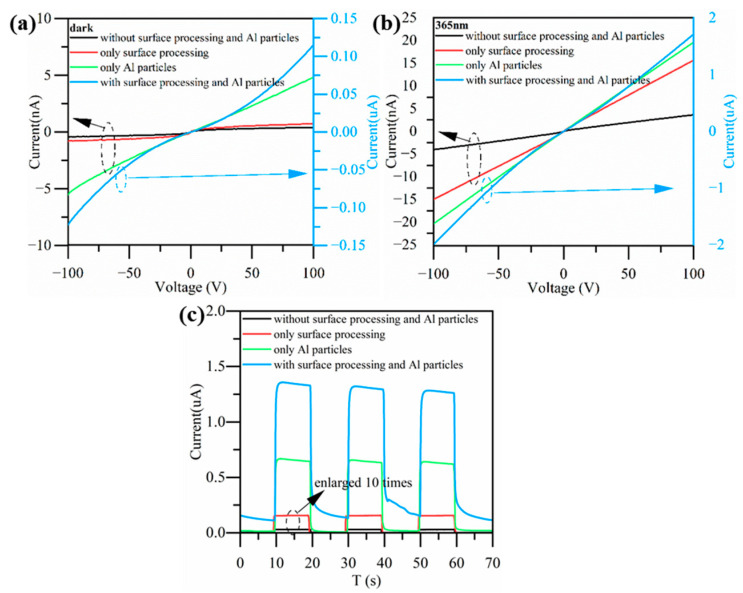
I-V characteristics of devices (**a**) in the dark, and (**b**) under 365 nm illumination. (**c**) I-T characteristics of devices at 100 V, part of the data was enlarged by 10 times for observation.

Based on the experimental results, surface processing was combined with Al nanoparticles to further improve the photo-detection performance of the device. A sputtering power of 100 W and sputtering time of 30 s were chosen for the Al nanoparticles sputtering. Additionally, a surface processing time of 40 s was employed. Figure 8a,b show the I-V characteristics of the device in the dark and under 365 nm illumination. Compared to the device with surface processing only or the device integrated with Al nanoparticles only, the device that combined the surface processing and Al nanoparticles had the highest photocurrent. This met our expectations. Figure 8c shows the I-T characteristics of the device at 100 V. It can be seen that the response time increased to a certain extent after introducing the Al nanoparticles. Table 2 describes the responsivity and response time of each kind of device at a voltage of 100 V. Compared to the unprocessed ZnO photodetector, the device that combined Al particles and surface processing could achieve a maximum response improvement of 848 times.

**Figure 9:**
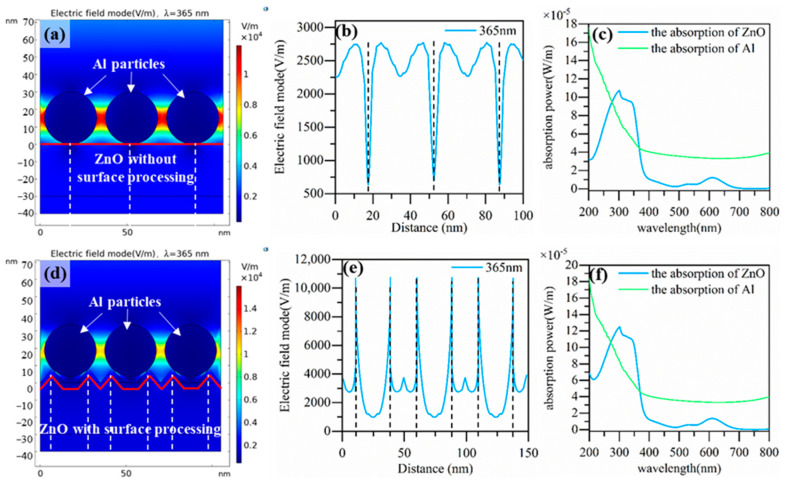
The effect of surface furrows caused by surface processing on LSP effect of Al nanoparticles. (**a**) No surface furrow structure and electric field distribution around the Al nanoparticles. (**b**) The electric field on the surface of ZnO along the red line, and (**c**) the absorption spectra of ZnO and Al for the structure with a flat surface. The dashed lines in (**a**,**b**) mark the same location, respectively. (**d**) ZnO surface with furrows and electric field distribution around the nanoparticles. (**e**) The electric field on the surface along the red line, and (**f**) the absorption spectra of ZnO and Al for the structure with an undulating surface. The dashed lines in (**d**,**e**) mark the same location, respectively.

According to the AFM characterization, it was found that surface processing for 40 s resulted in a roughness change of about 10 nm on the surface of the ZnO. The surface would have furrows within the range of 10 nm after the surface processing. These surface furrows would affect the distribution of the Al nanoparticles. Then, the LSP effect and coupling among the Al nanoparticles would be influenced accordingly. We used a simulation to illustrate the influence of surface undulation on the LSP effect and enhanced absorption. We used triangles with a height of 10 nm to mimic the surface furrows shown in Figure 9d. Figure 9a,d show the distribution of the electric field around the nanoparticles without and with ZnO surface furrows, respectively. Figure 9b,e show the electric field at the interface of the ZnO, indicated by the red line for the two different surfaces. Figure 9c,f show the absorption spectra corresponding to the two structures. It can be seen from the simulation results that the electric field at the interface between the ZnO and Al nanoparticles was significantly enhanced after the introduction of surface furrows. Additionally, the absorption of ZnO and Al was also enhanced. These simulation results explain well that an increase in the surface roughness would strengthen the LSP effect and enhance the absorption; therefore, the photo-response was improved by combining the Al nanoparticles with the surface processing. It was rather interesting that the responsivity enhancement resulting from the Al nanoparticles’ LSP effect could be further improved after introducing an appropriate roughness of the surface.

## 4. Conclusions

In this work, we systematically studied the effect of semiconductor surface roughness and morphology on the SPR enhancement of Al nanoparticles. Mechanical surface polishing was used to introduce a certain roughness on the surface of ZnO. We investigated the relationship between the surface roughness and surface processing time. More importantly, we found that introducing a certain roughness could improve the PD due to the increasing optical path length and stronger light scattering. Then, sputtering was employed to fabricate Al nanoparticles. The size and spacing of these Al nanoparticles were adjusted by changing the sputtering power and sputtering time. The experimental results showed that increasing the sputtering power and sputtering time within a certain range would increase the size of the Al nanoparticles. It enhanced the LSP effect and absorption, thus improving the device responsivity. However, we needed to prevent the Al nanoparticles from forming a continuous film, which could cause a short-circuit problem when choosing the sputtering power and time. Finally, we combined surface processing with Al nanoparticles. We found that introducing a certain surface roughness would enhance the electric field at the interface and coupling among the Al nanoparticles; thus, the SPR effect and absorption would be further enhanced. Compared to the ZnO PD, a nearly three orders of magnitude enhancement in the responsivity could be achieved by combining surface processing with Al nanoparticles. This work successfully improved responsivity by introducing surface roughness to enhance the LSP effect brought about by subwavelength metallic nanoparticles. It provides a new tuning dimension for SPR-enhanced PDs with metallic nanoparticles. Additionally, surface roughness can change in a large range, from atomically flat 2D graphene to a mechanically or chemically fabricated rough surface with roughness in micrometers. This indicates that we may tune SPR in a large range, and more underlying physics will be unveiled through further research. This also enables further enhancements for photodetectors based on nanoparticles, which might be a candidate for single-photon detection and quantum encrypted communication.

## Figures and Tables

**Table 1:** Surface roughness of ZnO under different surface processing parameters.

Surface Processing Time (s)	Surface Roughness (Ra/nm)	Average Thickness of ZnO (nm)
0	1.7	390.6
20	4.8	224.6
40	7.1	146.3
60	2.5	76.9

**Table 2:** Responsiveness and response time of devices after surface processing combined with Al particles (V = 100 V, P = 0.2 mW/cm^2^).

Device	Dark Current (nA)	Photocurrent (nA)	Responsivity (A/W)	Rising Time (s)	Decay Time (s)
Without surface processing and Al nanoparticles	0.42	3.64	7.41 × 10^−3^	0.08	0.1
Only surface processing	0.72	15.7	3.45 × 10^−2^	0.1	0.24
With Al nanoparticles	4.83	1.57 × 10^3^	3.61	0.24	0.16
Both surface processing and Al nanoparticles	1.45 × 10^2^	2.10 × 10^3^	6.28	0.34	1.1

## Data Availability

The data related with this work are not publicly available but can be obtained upon reasonable request from the corresponding author.
